# On the bulk degradation of yttria-stabilized nanocrystalline zirconia dental implant abutments: an electron backscatter diffraction study

**DOI:** 10.1007/s10856-017-5927-2

**Published:** 2017-07-06

**Authors:** V. Ocelík, U. Schepke, H. Haji Rasoul, M. S. Cune, J. Th. M. De Hosson

**Affiliations:** 1 Department of Applied Physics, Zernike Institute for Advanced Materials, University of Groningen, Nijenborgh 4, 9747 AG Groningen, Netherlands; 2 Department of Fixed and Removable Prosthodontics and Biomaterials, Center for Dentistry and Oral Hygiene, University of Groningen, University Medical Center Groningen, A. Deusinglaan 1, 9713 AV Groningen, Netherlands

## Abstract

**Abstract:**

Degradation of yttria-stabilized zirconia dental implants abutments due to the tetragonal to monoclinic phase transformation was studied in detail by microstructural characterization using Electron Back Scatter Diffraction (EBSD). The amount and distribution of the monoclinic phase, the grain-size distribution and crystallographic orientations between tetragonal and monoclinic crystals in 3 mol.% yttria-stabilized polycrystalline zirconia (3Y-TZP) were determined in two different types of nano-crystalline dental abutments, even for grains smaller than 400 nm. An important and novel conclusion is that no substantial bulk degradation of 3Y-TZP dental implant abutments was detected after 1 year of clinical use.

**Graphical abstract:**

## Introduction

Zirconia (ZrO_2_) belongs to an important class of industrial ceramic materials for structural applications due to its mechanical performance, e.g. high toughness and phase transformations [1]. Pure zirconia appears in three crystallographic modifications: between melting and 2370 °C the cubic phase is stable. Further cooling leads to the transformation to the tetragonal phase (t-ZrO_2_) and below 1170 °C the highly distorted monoclinic phase (m-ZrO_2_) is thermodynamically preferred. In fact, the use in practice of all three phases of zirconia is rather rare. The most structural engineering and biomedical applications utilize the tetragonal and cubic phases. The fact these phases are not stable at low temperatures requires zirconia doping with oxides such as Y_2_O_3_ which stabilize the high-temperature phases at room temperature or at temperature of human body. The stabilization effect is due to the oxygen vacancy concentration which originates from the introduction of Y_2_O_3_ to ZrO_2_ [2]. Another way of stabilizing the tetragonal structure at room temperature is the formation of nano-crystalline specimens in a sintered form [1]. To obtain powders of dense compacts at room temperature, the material has to contain crystals or grains below a certain critical size.

Stabilization of tetragonal phase has considerable consequences for both the mechanical and electrical properties of zirconia. The tetragonal phase is preferred when a high toughness is required. Within the field of dentistry Y_2_O_3_ stabilized ZrO_2_ is used as a ground material for indirect restorations (i.e. crowns and bridges), for dental implants and implant abutments [3]. Y_2_O_3_ stabilized ZrO_2_ has a white appearance and can be colored easily which is, in contrast to metals, of an aesthetic advantage [4]. Also, its use allows CAD-CAM production of dental restorations [5]. However, the lifetime of widely used 3 mol.% yttria-stabilized tetragonal zirconia poly-crystals (3Y-TZP) for these biomedical applications is rather unknown and, is predominantly estimated on the basis of in vitro accelerated ageing experiments [6]. Here, the intraoral conditions during clinical use are poorly mimicked, resulting in a doubtful and questionable external validity. In fact, it is a major source of concern.

The structurally detrimental phase transformation due to spontaneous increase of the amount of the brittle monoclinic phase is known as low-temperature degradation (LTD) [7], because this aging process of the material occurs at moderate temperatures below 400 °C. The influence of water or water vapor on this transformation was also studied, see [8]. However, the kinetics, rate constants and other details such as the possible difference between the attack by water vapor and liquid water remain unknown.

**Fig. 1:**
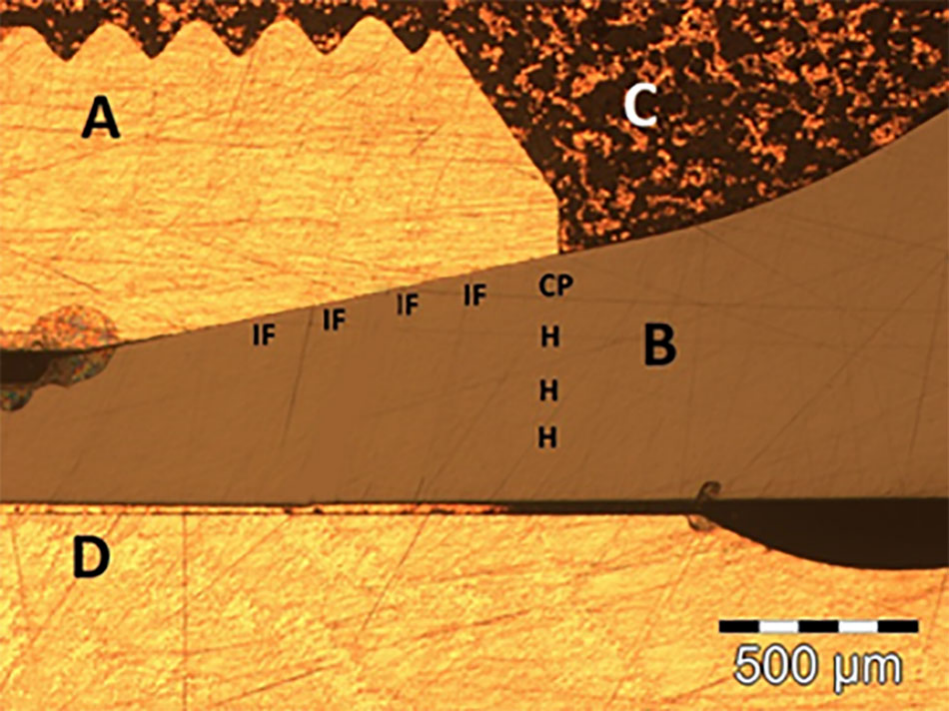
Optical microscopy image from one part of 3Y-TZP embedded abutment (area B) before its final grinding and polishing. A—external thread from Ti alloy; B–cross section of embedded abutment; C—copper based conductive mount; D—inner screw of dental implant; CP, IF and H—approximate locations of EBSD scans

This study is aimed at determining in detail whether the currently used polycrystalline yttria stabilized tetragonal zirconia (3Y-TZP) dental implant abutments are susceptible to LTD at temperature of human body in a clinical setting (i.e. screw retained to a titanium implant on the bone-side and bonded to a full crown on the oral side). A small surface area between the connecting parts was constantly in contact with the oral environment. The location of the EBSD analysis was chosen according to assumed stress peaks inside the material (Fig. 1).

In particular Electron Backscatter Diffraction is explored in the structural analysis of these phenomena since its resolution is of the order of 40–50 nanometers [9]. In addition this method provides access to crystal orientation correlations, representation of texture and grain boundary character distributions via grain orientation maps. Samples of 3Y-TZP dental abutments were removed, after 1 year of clinical use, and examined on t-m transformation. Two types of zirconia sintered dental abutments were tested: Atlantis^TM^ CAD-CAM designed and ZirDesign^TM^ stock specimen, both from one and the same producer (Dentsply Sirona Implants, Mölndal, Sweden). Pristine, geometrically identical samples were conserved in a dry environment at room temperature for pairwise comparison. The amount of m-ZrO_2_ phase was determined using electron backscatter diffraction (EBSD). Experimental methods to study phase transformations in ZrO_2_ with a focus mainly on hip prostheses are summarized in [7]. Traditionally this type of study has been carried out using neutron diffraction and/or electron diffraction in a transmission electron microscopy (TEM). The most extensively used is X-ray diffraction (XRD) [10]. The latter method is a fast and a non-destructive method, allowing the monitoring of LTD evolution on the same specimen. Nonetheless, XRD is limited as far as the lateral resolution is concerned and the typical spot size is in the order of millimeters. X-ray powder diffraction is best for identification of homogeneous and single phase materials. It is also not very precise for a monoclinic phase content lower than 5%, making it not suitable for monitoring the onset of the transformation [7].

## Experimental and EBSD data handling

Four samples were retrieved from 4 patients who were treated for premolar replacement at the Center for Dentistry and Oral Hygiene of the University Medical Center Groningen; the Netherlands. These patients received a single crown on a dental implant. The dental abutments were used to connect the dental implants and the crowns. Two types of dental abutments were used, standard stock abutments (ZirDesign) and individually computer aided designed and manufactured abutments (Atlantis). After one year of clinical use the dental abutments were replaced. The control group consists of 4 pristine dental abutments of the same types, with the same anatomy, preserved in a dry environment at room temperature during the same time as for clinical samples.

Sample preparation procedure for microstructural observations consists of filling the sample holes using conductive Demotec70 epoxy. After drying, the samples were embedded using a Buehler hot mount with Allied Copper-based conductive mounting powder. Grinding with SiC 180 grade powder under water was used until the required cross section was exposed. Further grinding using Struers Piano 220 and 1200 magnetic disks was performed before lapping by 9, 6, 3 and 1 µm diamond particle suspensions (each about 5 min). Final polishing by Struers OPS silica suspension (40 nm) for 10 min was used to prepare surface of zirconia implants for EBSD observation.

EBSD microstructural characterization was conducted using a Philips XL30 FEG ESEM microscope operated at standard high vacuum mode (5.5 × 10^−5^ mBar) equipped with TSL EDAX Electron Backscatter Diffraction system containing DigiView 3 CCD camera.

In total sixteen scans were collected from each abutment sample. The specimens were embedded in such a way that the buccal and the lingual side of the implant abutment could be scanned. Because of the time consuming scanning procedure, the following areas, shown in Fig. 1 were selected for EBSD scanning:(I) Contact point (CP) between the dental abutment and the dental implant, as this area is assumable the area of the highest stress concentration built-up during the service; (II) Four areas at the interface along the dental implant (IF) in the apical direction, and (III) Three areas (H) from contact point towards the axis of the dental implant. The CP were scans with size of 25 × 25 µm as close as possible to the dental implant (~200 nm) with the distance between scanning points of 50 nm. Four IF scans were made, also as close as possible to the dental implant (~200 nm), with an equal distance between the scan areas of 100 µm. Three H scans were made with an equal distance of 100 µm between them and as close as possible to the CP scan. The IF and H scans are 5 × 5 µm in size with distance between scanning points of 40 nm. Hexagonal grid of point’s arrangement was selected for all scans. Data are presented in different types of orientation imaging microscopy (OIM) maps, showing grain orientations, type of phase and grain boundaries.

EDAX TSL OIM Data Collection software v7.3 was used for the collecting of OIM data. The electron beam acceleration voltage was set to 10 kV with the beam current of 2 nA. This beam setting suppresses a tendency of the non-conductive ceramic sample to charge and distort collected EBSD maps and also allows achieving higher lateral resolution required for samples with small grains (~500 nm). CCD camera pattern binning of 8 × 8 allows the EBSD patterns collection and indexing speed of 70 fps resulting in a final time for one EBSD scan of 70 min for large scan of 25 × 25 µm size and about 4–5 min for scan with size of 5 × 5 µm, respectively.

Due to the absence of the tetragonal and monoclinic ZrO_2_ phase files in the standard TSL database new phase files were constructed on the base of detailed crystallographic information [11] in COD files #2300296 and #2300297 for monoclinic and tetragonal phase, respectively. Lists of main reflectors were carefully tuned on high quality EBSD patterns collected for a few different crystal orientations for both phases.

**Fig. 2:**
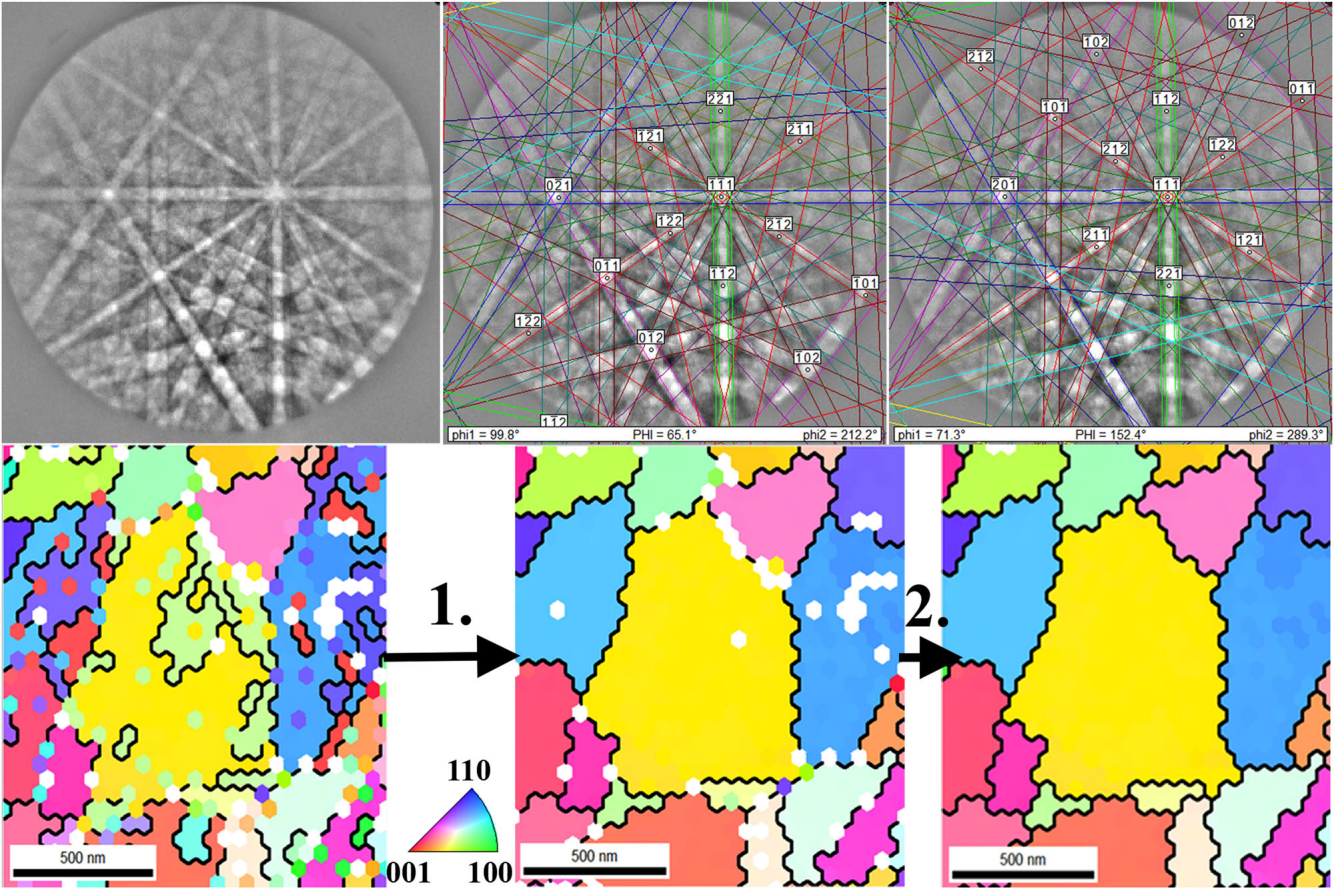
Top row from *left* to *right*: typical EBSD pattern of t-ZrO_2_ and its two possible indexing with a close number of votes. *Bottom* row: Local view on [001] Inverse Pole Figure map before and after data cleaning procedures. From *left* to *right*: raw IPF map, IPF map after pseudo-symmetry cleaning (1.), IPF map after Confidence Index standardization and neighbor orientation correlation cleaning (2.). Grain boundaries (>5°) are marked by *black lines*

Unfortunately, typical pseudo-symmetric mis-indexing feature were present on orientation maps of tetragonal ZrO_2_ phase connected with the fact that for some crystal orientations a few very close indexing solutions are possible as demonstrated in the upper part of Fig. 2, where two automatic indexing solutions with two different crystal orientations with the same number of votes were detected. Figure 2 also demonstrates a high quality of Kikuchi patterns collected on t-ZrO_2_ phase crystals. Bottom part of this figure shows from left to right (I) detail of [001] inverse pole figure (IPF) map of raw data, (II) the same map after pseudo-symmetry cleaning (90° @ [110], tolerance angle 1°) and finally (III) the same map after last two cleaning steps, respectively. These steps consist of grain Confidence Index (CI) standardization and Neighbor Orientation Correlation step. Typically, the first pseudo-symmetry cleaning step changes the orientation of about 25% of t-ZrO_2_ points. Grain CI standardization cleaning procedure just unified the CI parameter for all points inside one grain and sets it to the maximum observed number for that particular grain. During this cleaning step there is no change in crystal orientations of individual points, just the probability of proper indexing of individual points inside one grain is equalized to its maximum for a particular grain. This is important for the third cleaning step, in which the orientations of the isolated non-indexed points or points with low CI are changed to the orientation of their neighbors, if these have the same orientation with high CI. Figure 2 shows a typical result of these two cleaning steps applied after pseudo-symmetry cleaning. It is clear that mainly isolated points on the boundaries between two or three grains are corrected and the whole map does not change its character.

One should note that pseudo-symmetry cleaning (sometimes also called as a “simple” harmonization of grain orientation [12]) is a considerable intervention into the t-ZrO_2_ crystal orientation data, which on one side improves the visual appealing of the grain orientation maps and grain size distributions, but it should not be used in characterization of grain or phase boundaries.

## Results

**Fig. 3:**
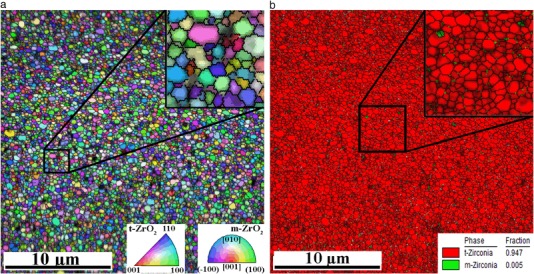
[001] IPF map (*left*) and phase map (*right*) combined with IQ map of 1 year used Atlantis sample at CP location. Grain boundaries (>5°) are shown as *black lines*. Scan area is 25 × 25 µm

Figure 3 shows typical [001] Inverse pole figure (IPF) map and Phase map collected on the Atlantis CAD-CAM sample that had functioned clinically for one year. Maps show the same CP sample area with size of 25 × 25 µm, containing about 290 k points with distance of 50 nm between them. More than 99.8% points were successfully indexed during collection of EBSD map, but only points with CI larger than 0.05 are colored. Both maps are combined with Image Quality (IQ) map (pattern contrast map) represented by points brightness. From both insets located at top right corners one may conclude that the IQ parameter is higher at the centers of t-ZrO_2_ grains and decreases slightly at tetragonal phase grain boundaries but substantially inside monoclinic phase. Tetragonal grains have sub-micrometer size and monoclinic phase grains are even smaller.

**Fig. 4:**
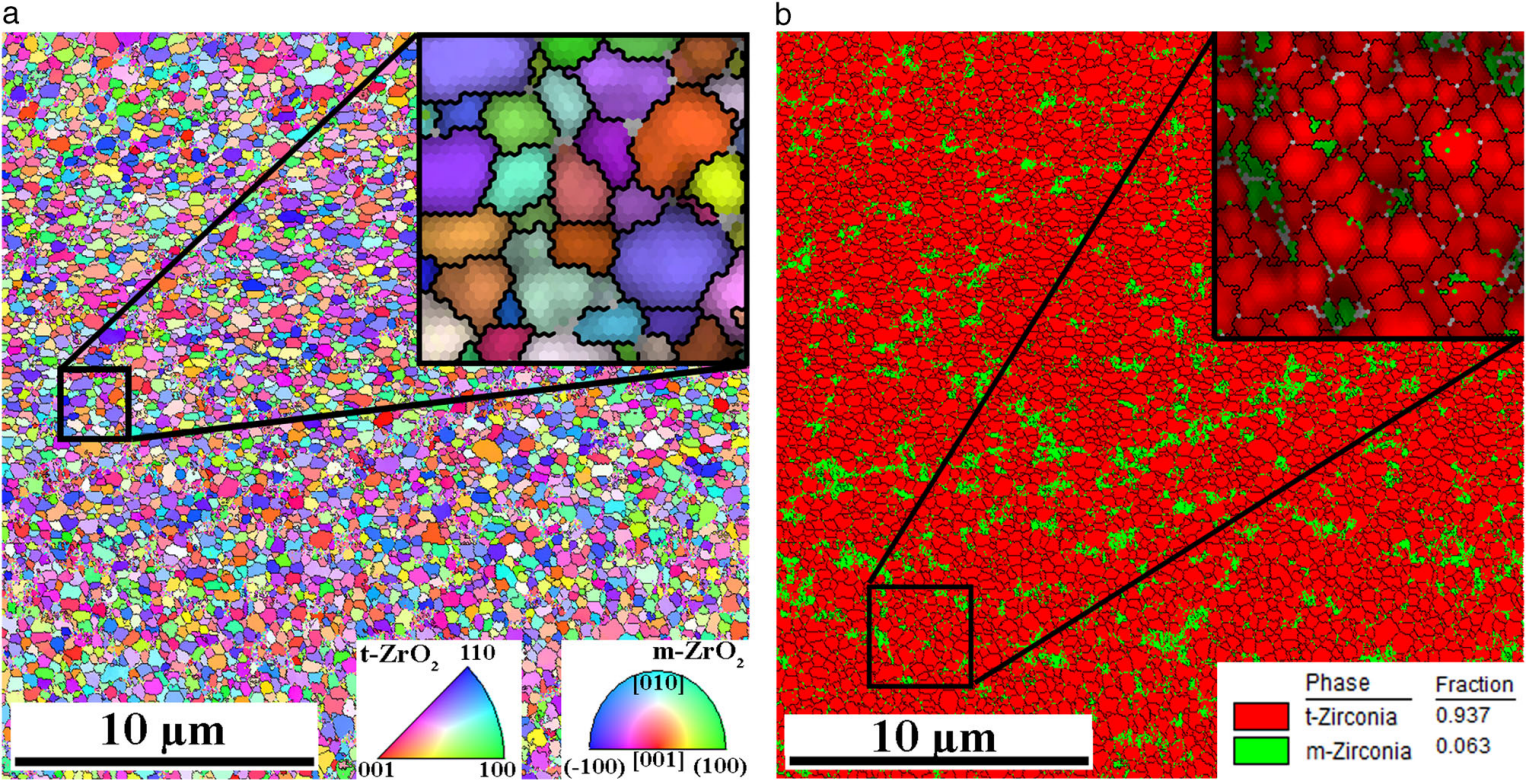
[001] IPF map (*left*) and phase map (*right*) of 1 year used ZirDesign sample at CP location. Grain boundaries ( > 5°) are shown as *black lines*. Scanning area is 25 × 25 µm

As Fig. 3 clearly demonstrates, this one year clinically tested Atlantis sample contains less than 1% of monoclinic phase. For a comparison Fig. 4 shows the IPF and phase maps for ZirDesign sample also after one year of clinical use. In this case more than 6% of monoclinic ZrO_2_ phase was detected.

Details of phase maps on both previous figures clearly demonstrate that m-ZrO_2_ phase nucleates at grain boundaries and on triple points of tetragonal grains. Table 1 summarizes average amount of tetragonal phase detected in both type of samples and also compares it between used and not used abutments. Averaging has been made over all 16 locations on two samples tested in each group. Ninety five percent confidence intervals of mean are also indicated in Table 1. It has to be noted that main source of variance has been observed between two samples and not between different places of EBSD scans indicated in Fig. 1.

**Table 1:** Comparison of the mean value of content of monoclinic ZrO_2_ phase in used and not used states for both type of abutments. 95% of confidence intervals for mean values are also indicated

Sample	Not used		Used	
	Mean [%]	Error	Mean [%]	Error
Atlantis	0.57	±0.27	1.0	±0.27
ZirDesign	6.17	±1.21	3.8	±1.30

Statistical testing rejected the hypothesis about influence of 1 year clinical use of abutment on the amount of monoclinic phase for both type of materials. The negative results were also obtained when a hypothesis of influence of different sample place on the amount of observed monoclinic phase was tested. Therefore a general conclusion could be made that the amount of monoclinic phase does not increase in both type of tested abutments during one year of clinical use.

**Fig. 5:**
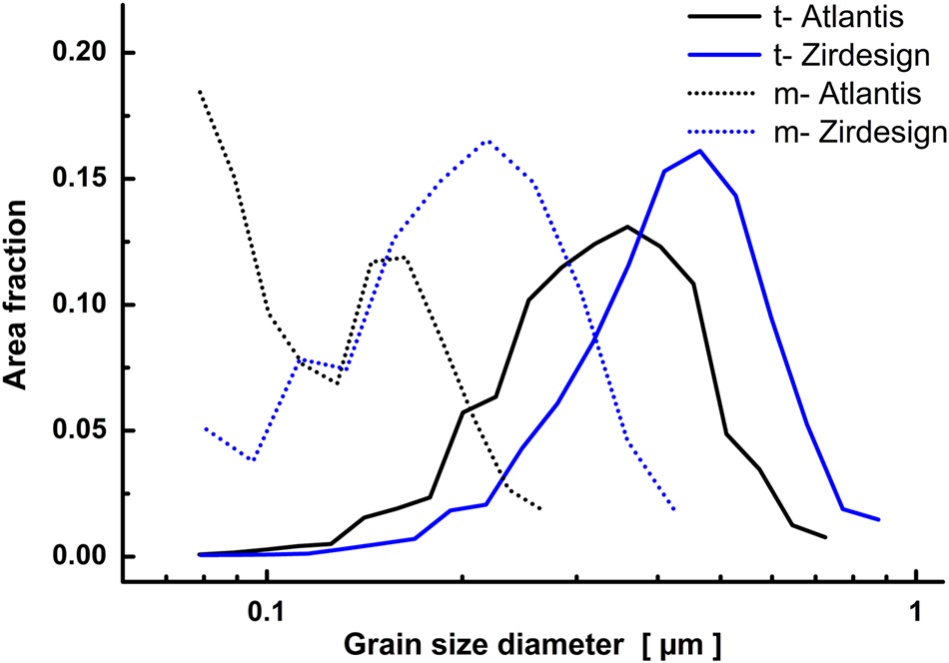
Grain size distributions calculated for tetragonal and monoclinic grains (misorientation >5°) of both type of specimens

Figure 5 compares grain size distributions calculated from OIM maps for both phases in these two types of samples. ZirDesign samples show a larger average grain size of tetragonal phase (410 nm) in comparison with Atlantis abutments (340 nm). Grain size distributions of tetragonal grains for both type samples are very close to a log-normal distribution with a little bit larger dispersion in Atlantis material. The average size of monoclinic phase grains is only a fraction from the size of the tetragonal phase, which confirms the fact that monoclinic phase nucleates at grain boundaries and the triple points between tetragonal grains and propagates into them, as seen in the phase map insets in Figs. 3 and 4.

**Fig. 6:**
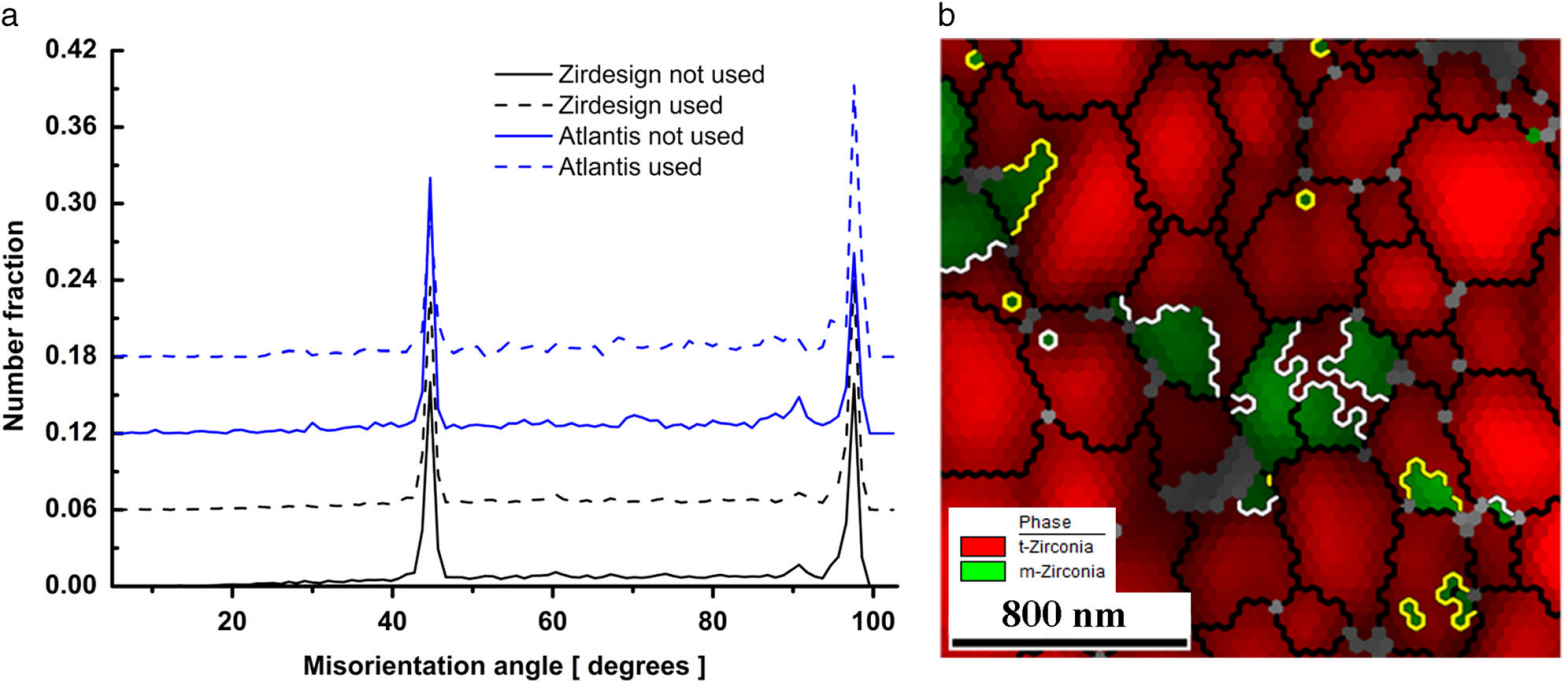
*Left*: Distribution of misorientation angles between tetragonal and monoclinic grains detected in all type of samples. *Right*: Detail of Phase map combined with IQ map and grain boundaries observed on ZirDesign sample using 25 × 25 µm OIM scan. Grain boundaries with misorientation larger than 5° are marked by *black lines*. Grain boundaries with special misorientation angles are highlighted by *color lines*: 97.5 ± 1 degree misorientation boundaries are marked in *white* and 44.6 ± 1 degree boundaries in *yellow* color, respectively (color figure online)

Crystal orientation data collected during EBSD scanning allows a more detailed study of tetragonal to monoclinic phase transformation. A special distribution of misorientation angles between neighboring tetragonal and monoclinic grains was detected on all samples and it is shown on the left part of Fig. 6. Two special misorientation angles with a high presence rate were detected with maxima at 44.6 and 97.5 degrees. The right side of Fig. 6 shows a detailed part of the microstructure of ZirDesign sample. The phase map is combined with IQ map and grain boundaries are highlighted by lines. General grain boundaries (>5°) are *black*, whereas grain boundaries with special angles shown on left side of the figure are marked by *yellow* and *white* color, respectively. Figure 6 demonstrates clearly as was observed on all four types of samples: almost every monoclinic ZrO_2_ grain has just one tetragonal ZrO_2_ neighboring grain with the misorientation angle of 44.6 ± 1 or 97.5 ± 1 degrees. Assuming that a transformation from tetragonal to monoclinic phase occurred, a relation between parent and daughter grain becomes evident. Monoclinic grain grew mainly from triple points of t-ZrO_2_ grain boundaries and proceeded into the parent t-ZrO_2_ grain.

**Fig. 7:**
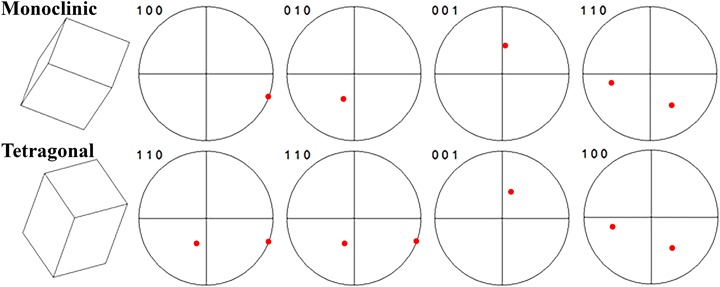
Pole figure plots of different planes for daughter (monoclinic) and parent (tetragonal) grains having misorientation angle of 46.4° for demonstration OR (1)

The sharp misorientation angles detected between parent t-ZrO_2_ and daughter m-ZrO_2_ phase indicate the existence of crystallographic orientation relationship(s) (ORs) associated with t-m transformation of 3Y-TZP phase. The character of these relationship have been revealed by observing a substantial amount of mutual pole figure plots of parent tetragonal and daughter monoclinic grain having one of these two misorientation angles. A pole figure is a graphical representation of the orientation of crystal in space. Stereographic projection is used to plot poles—the intersections of selected crystal planes normal with a sphere. Figure 7 compares pole figure plots for parent and daughter grain having misorientation angle of 44.6 degrees. On more than 15 such pairs the same OR has been observed for all pairs having this smaller misorientation angle:

1 $$\begin{array}{l}\\ 	 {\left[ {{\rm{100}}} \right]_{\rm{m}}}\left\| {{{\left[ {{\rm{110}}} \right]}_{\rm{t}}}\,{\rm{and}}\,{{{\rm{[010]}}}_{\rm{m}}}} \right\|{{\rm{[110]}}_{\rm{t}}}\; {\rm{and}}\;{{\rm{[001]}}_{\rm{m}}}{\|_{\rm{c}}}{{{\rm{[001]}}}_{\rm{t}}}\; \\ \\ 	 {\rm{and}}\;{{{\rm{(100)}}}_{\rm{m}}} \left\|\right.{{\rm{(100)}}_{\rm{t}}}\\ \end{array}$$

where || means that directions or planes are parallel and ||_c_ denotes they are almost parallel. The average of small angle between [001] directions in the coupled tetragonal and monoclinic crystals were measured as 8.8° with standard deviation of 0.6°. This value is in agreement with the inclination of unit cell angle β from the perpendicular direction, being 99.215° for monoclinic ZrO_2_ phase [13].

However, for monoclinic and tetragonal parent–daughter crystal pairs with misorientation angle of 97.5° situation is more complicated and one of the following two different variants of ORs was detected:

2 $$\begin{array}{l}\\ 	 {\left( {100} \right)_{\rm{m}}}\left\| {{{\left( {110} \right)}_{\rm{t}}}\,{\rm{and}}\,{{\left[ {010} \right]}_{\rm{m}}}} \right\|{\left[ {001} \right]_{\rm{t}}}\,\\ \\ 	 {\rm{and}}\,{\left( {001} \right)_{\rm{m}}}{\| {_{\rm{c}}{{\left( {110} \right)}_{\rm{t}}}\,{\rm{and}}\,{{\left( {101} \right)}_{\rm{m}}}} \|_{\rm{c}}}{\left( {010} \right)_{\rm{t}}}\\ \end{array}$$

3 $$\begin{array}{l}\\ 	 {{\rm{(100)}}_{\rm{m}}}\left\| {{{{\rm{(001)}}}_{\rm{t}}}\;{\rm{and}}\;{{{\rm{[010]}}}_{\rm{m}}}} \right\|{{\rm{[110]}}_{\rm{t}}}\;\\ \\ 	 {\rm{and}}\;{{\rm{(001)}}_{\rm{m}}}{\| {_{\rm{c}}{{{\rm{(110)}}}_{\rm{t}}}\;{\rm{and}}\;{{{\rm{(011)}}}_{\rm{m}}}}\|_{\rm{c}}}{{\rm{(100)}}_{\rm{t}}}\\ \end{array}$$

**Fig. 8:**
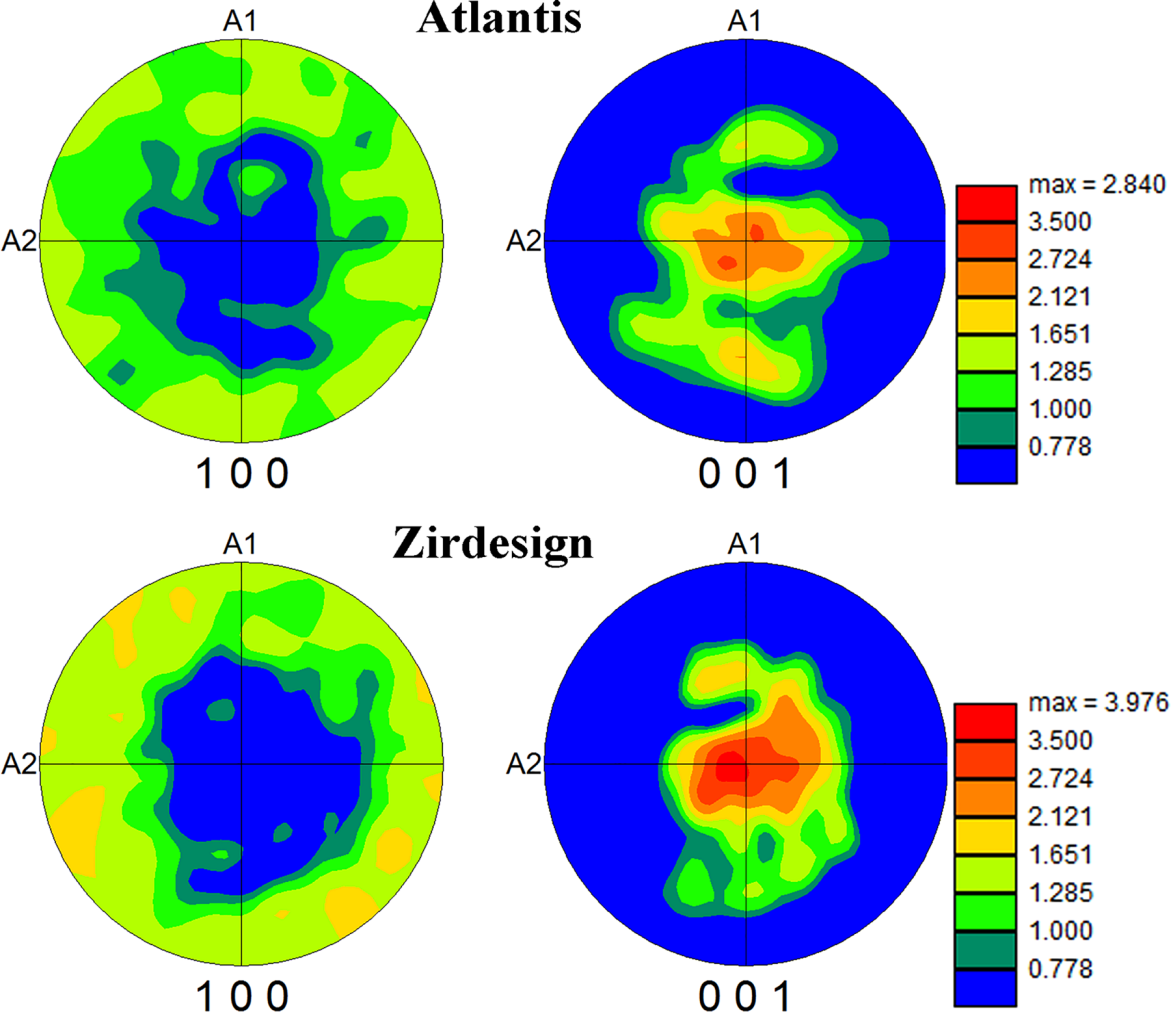
(100) and (001) Pole figure texture plots of tetragonal phase calculated from EBSD scans performed on Atlantis and ZirDesign 3Y-TZP dental abutments

Finally the texture (preferred crystal orientation in the sample) detected on both type of dental abutments was examined in detail. The textures observed are shown in Fig. 8 for both types of abutments in the form of texture plots. A texture plot is a type of pole figure plot in which all detected crystal directions are shown in a single figure, i.e. in the form of local density of poles. Random texture should result in a uniform density of poles. On the contrary, texture plots of tetragonal ZrO_2_ grains shown in Fig. 8 are both characterized by a preferential orientation of the (001) plane normal parallel to the samples surface normal *A3*, which is in accordance to the tangential direction towards rotation axes symmetry of the dental abutment. This texture is slightly stronger in the ZirDesign sample, expressed by higher value (3.98) of multiples of uniform density 1 in (001) texture plot.

## Discussion

From the observations shown in Figs. 3b and 4b it is concluded that careful EBSD mapping could be used to measure a rather small amount of monoclinic phase in nano-crystalline 3Y-TZP dental abutments. An attempt to confirm this EBSD observed m-ZrO_2_ phase amounts in the same samples by standard XRD diffraction measurement according [10] failed due to lack of sensitivity of detecting of monoclinic phase peaks in the diffraction spectrum. However, from the results shown in Table 1 one may conclude that in general ZirDesign abutments contain more m-ZrO_2_ phase than Atlantis ones. However, all measured amounts of monoclinic phase in our experiment were far below the value when a degradation of mechanical properties is expected. The amount of monoclinic phase measured during accelerated hot steam test [10] start to exceed 10% after a few hours at increased temperature and authors suggested to use optical interferometry and atomic force microscopy for the initial stage of transformation, when small amounts of monoclinic phase should be detected. Our results confirmed that EBSD is another appropriate method with an advantage of crystallographic information. Due to very careful sample preparation consisting of gradual grinding and polishing we do not expect that the detected monoclinic phase could develop during this procedure.

Smaller amounts of the monoclinic phase in Atlantis material (Table 1) could be related to its smaller grain size (Fig. 5). It is known that a smaller grain size enhances the stability of the tetragonal phase [7]. The experimentally observed lack of increase of amount monoclinic phase content in the specimens was indirectly confirmed by the fact, that also no substantial change of grain size distribution between used and not-used sample was found. An observable shift towards a smaller grain distribution was observed by EBSD on artificially aged ZrO_2_ samples in autoclave [14], which is obvious when only a small part of tetragonal grain transforms to monoclinic form.

Simultaneous EBSD mapping observations of different crystal modifications inside an MgO stabilized zirconia particles embedded in steel matrix were already observed in [15]. Unfortunately, no information about crystallographic orientation relationships between them has been provided. On the base of theoretically predicted possible orientation relationships between parent tetragonal and daughter monoclinic ZrO_2_ phase [16], Cayron et al. [17] were able to reconstruct orientations of primary tetragonal grains inside the fully transformed monoclinic microstructure. Orientation relationships between parent tetragonal and daughter monoclinic phase suggest that the transformation has a martensitic character although not only ORs predicted from a simple lattice distortions [16] were detected.

Relatively strong texture of tetragonal phase observed in both type of samples (Fig. 8) is in contradiction with the recently published EBSD observations [18] on commercial hipped dental 3Y-TZP ceramics, where no texture was reported. However, in comparison with our results (Figs. 3 and 4) a relatively low quality OIM map was presented in [18], and no information is provided concerning the characterization of the tetragonal crystal phase used for indexing of the Kikuchi patterns. Because the authors did not mention any complexity with pseudo-symmetry during automatic indexing, we suspect that the cubic instead of tetragonal phase was used for indexing and therefore the preferential orientation of an elongated tetragonal axis *c* could not be detected. In comparison with our results also a larger average grain size has been observed on level of 500 nm.

In this study we have clearly demonstrated that EBSD appears as the experimental means to bridge the gap between the very local transmission electron microscopy information and the volume averaging information that comes out from X-ray diffraction. It is shown that EBSD is an efficient way to study zirconia-based materials for biomedical applications, their stability and phase transformations. A possibility to realize EBSD observations using low-vacuum scanning electron microscopes [19] and the rapid scanning offered by the fast acquisition cameras [20] allow EBSD mapping of zirconia without any deleterious charging effects.

There are few studies in the literature where the EBSD approach was used to study the microstructure of 3Y-TZP [14, 21–24]. Often the drawbacks with charging effects are circumvented by adding a thin carbon coating or by lowering the SEM voltage during OIM scan. In this study we have found that on a well (mechanically) polished surface of 3Y-TZP the combination of SEM voltage reduction (10 kV) and fast OIM scanning (>60 fps) could result in mapping areas large enough for nanostructured materials (more than 5000 grains). High quality of Kikuchi pattern allows reliably distinguish tetragonal and monoclinic ZrO_2_ phase and also to study orientation relationship between them. However, complications due to pseudo-symmetry during automatic pattern indexing (similar as in [23, 25]) requires 90@ <110 > harmonization of grain orientation to obtain correct grain size distributions.

The fact that we did not observe an increasing concentration of monoclinic phase after one year of clinical test could be understood within the framework of long-time aging studies of tetragonal 3Y-TZP at temperature of human body [26, 27]. The phenomenon of tetragonal to monoclinic transformation as detected by X-ray micro-diffraction and confirmed by SEM observations is considered being due to the surface exposed to humidity. After one year the transformed layer attains a thickness less than 1 µm. However, our sample preparation procedure (mechanical polishing) does not allow studying areas very close to the outer surfaces exposed to the environmental conditions. Only 1–2 grains at the edge of our scans were directly exposed to the humid conditions and these are statistically less relevant in our observation. Ion beam cross-sectioning procedure [28] may result in a sharper sample edge allowing thus more reliable subsurface EBSD characterization and receiving simultaneous access to the surface vicinity and to the bulk information, and their comparison from the same specimen. Our measurements are therefore more relevant for a characterization of bulk microstructure of dental abutments mainly after their production. However, importantly our measurements confirm that bulk characteristics of 3Y-TZP are not changed during one year of clinical function.

## Conclusions

We have used electron backscatter diffraction in a scanning electron microscope to study the stability of yttria-stabilized ZrO_2_ dental implants abutments that had functioned clinically for one year. The overall conclusion is that EBSD turns out to be an excellent method to study the stability of ZrO_2_ dental implant abutments locally and to detect small amounts of the brittle m-ZrO_2_ phase. Moreover details of the microstructural characteristics such as grain size distribution, tetragonal and monoclinic phase crystal orientation relationships and technological texture are provided.

As far as the details are concerned we conclude that the average grain size of the tetragonal phase in the pristine ZirDesign samples was larger than the average grain size of the tetragonal phase in the pristine Atlantis implant abutments, being both well below 500 nm. Texture has been detected in both types of Atlantis and ZirDesign abutments. The preferred orientation of tetragonal ZrO_2_ nanocrystalline grains is induced in the manufacturing steps during abutment fabrication.

An important finding is that none of the samples showed an increasing volume fraction of the monoclinic phase after one year of clinical use. All measured values were far below 25%, the critical value for catastrophic embrittlement. The lowest measured amount of monoclinic phase was 0.5 vol.% and the highest 8.8 vol.%. The pristine ZirDesign dental implant abutments showed a higher average volume percentage of the monoclinic phase (6.44 vol.%) than the Atlantis (1.16 vol.%) dental abutments. Also, the EBSD scans at different scan areas did not reveal more monoclinic phase at critical places with expected stress concentrations.

It is fair to say that a disadvantage of EBSD is the destructive character of sample preparation damaging both the dental abutment and the attached artificial crown. This precludes a direct comparison of the same sample before and after clinical use. Also the nonconductive nature of Y-TZP caused charging during the EBSD scanning at “standard” conditions. This means that we had to make scans of smaller areas and use relatively high frame collection rate. Reducing the SEM acceleration voltage is also important. The 25 × 25 µm sized scans with 50 nm point spacing were possible without map distortion after reducing the voltage to 10 kV.
